# Portable Multi-Channel Electrochemical Device with Good Interaction and Wireless Connection for On-Site Testing

**DOI:** 10.3390/mi14010142

**Published:** 2023-01-05

**Authors:** Yifei Xu, Haohao Ning, Shixin Yu, Shikun Liu, Yan Zhang, Chunyan Niu, Yongzhuo Zhang, Sze Shin Low, Jingjing Liu

**Affiliations:** 1 School of Automation Engineering, Northeast Electric Power University, Jilin 132012, China; 2 Center for Advanced Measurement Science, National Institute of Metrology, Beijing 100029, China; 3 National Institute of Metrology, Beijing 100029, China; 4 Nottingham Ningbo China Beacons of Excellence Research and Innovation Institute, University of Nottingham Ningbo, Ningbo 315100, China

**Keywords:** on-site testing, portable, Narrowband Internet of Things, cloud processing

## Abstract

It is very important to rapidly test the key indicators of water in the field to fully evaluate the quality of the regional water environment. However, a high-resolution measuring device that can generate small currents for low-concentration analytes in water samples is often bulky, complex to operate, and difficult for data sharing. This work introduces a portable multi-channel electrochemical device with a small volume, good interaction, and data-sharing capabilities called PMCED. The PMCED provides an easy-to-operate graphical interactive interface to conveniently set the parameters for cyclic voltammetry or a differential pulse method performed by the four electrode channels. At the same time, the device, with a current sensitivity of 100 nA V^−1^, was applied to the detection of water samples with high background current and achieved a high-resolution measurement at low current levels. The PMCED uses the Narrow Band Internet of Things (NB-IoT) to meet the needs for uploading data to the cloud in remote areas. The electrochemical signal preprocessing and chemometrics models run in the cloud, and the final results are visualized on a web page, providing a remote access channel for on-site testing results.

## 1. Introduction

As an important means of obtaining the water conditions of the natural environment in a straightforward manner, on-site testing can provide information on water samples in a timely manner to reduce the changes in sample composition caused by transportation and storage [1,2]. However, expensive, time-consuming, and complicated operation steps are the challenges faced by on-site water quality testing. The time-consuming traditional detection method is difficult to ensure a high detection frequency, which limits the time scale for on-site testing. At the same time, a bulky instrument that is inconvenient to move puts forward higher requirements on the detection conditions, and it is difficult to cover a wider range on the spatial scale. The development of a portable instrument that is suitable for on-site testing, simpler to operate, and faster at detecting has gradually attracted attention [3,4].

The electrochemical analysis method can quickly determine the concentration of a target analyte with a simple operation. At the same time, the concise potential excitation and current acquisition methods of electrochemical approaches can be integrated into portable devices to achieve similar performance with traditional electrochemical workstations [5,6,7]. With screen-printed electrodes, the electrochemical analysis method is very suitable for on-site testing with low cost and good portability [8,9]. In the early days, miniaturization and low power consumption were the main directions of most battery-powered portable electrochemical detection devices, which have made progress on the basis of the development of integrated circuits and system chip components. Then, the portable electrochemical detection device was developed to connect with smart devices, such as smartphones, and their powerful network connection function was utilized to access the cloud server for data transmission and sharing [10,11,12]. These designs have advantages in terms of device portability and data management. By comparison, it is a more suitable strategy for on-site testing to combine a good interaction mode and remote access capability of portable devices without relying on additional smart devices so that portable electrochemical detection devices can maintain full flexibility and autonomy for on-site testing [13,14,15]. A graphical user interface simplifies the operation of the device with a friendly interface design [16]. It can improve the efficiency of information interaction by taking up relatively few resources of portable devices. In terms of wireless communication, the Narrowband Internet of Things (NB-IoT) is a suitable wide-area wireless technology that can provide remote access capability [17,18,19]. NB-IoT has a strong coverage capability and can provide a reliable connection in remote places to transmit field detection data. At the same time, it can maintain low power consumption, increasing its working time under limited resources [20]. Remote access to the key indicators of water at on-site testing can enable remote managers to obtain timely sample testing information and make decisions. A cloud server can provide uninterrupted data management and access services for this demand [21,22,23,24]. At the same time, the electrochemical signal analysis, processing, and calculation of the stoichiometric model can also be efficiently completed in the cloud server to reduce the computing pressure on portable devices.

A high background current exists in the water quality detection results due to a matrix effect. Therefore, a high-resolution electrochemical analysis device can measure the effective peak even under high background current. The water quality assessment of natural water requires on-site testing [25,26,27]. Water resource regulators try to maintain the COD_Mn_ and multiple water quality indicators for assessing the organic pollution level within a reasonable range so as to enhance the control of the state of water pollution [28,29,30], realizing the management of water resources.

In this study, a portable multi-channel electrochemical analysis device (PMCED) with good interactivity and wireless connectivity was designed, and a platform for the management, analysis, and visualization of electrochemical data was built in the cloud to jointly form an electrochemical analysis system suitable for on-site testing. The graphical interface in the portable device can flexibly set the parameters for performing cyclic voltammetry (CV) or differential pulse voltammetry (DPV) for electrochemical detection. The multiplexer provides four electrode channels. The integrated low-power Bluetooth and NB-IoT realize a short-distance wireless interaction and long-distance wireless data transmission, respectively. As a demonstration, the system is used in the field detection of COD_Mn_ in several natural water bodies. In water quality detection with a high background current, the device maintains a high resolution for a small response current. The PMCED, with a good interaction mode and wireless connection capability, fully ensures rapid on-site testing and wireless transmission of high-quality data. The obtained data is stored and analyzed on the cloud server. Finally, the visualization results for COD_Mn_ in each water area are displayed.

## 2. Design of the PMCED

**Figure 1:**
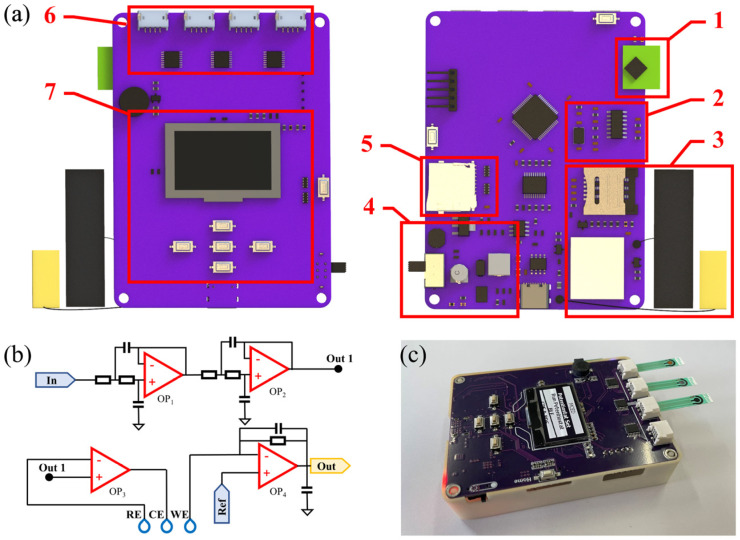
PMCED. (**a**) Each function module of the PMCED: 1. Low power Bluetooth module; 2. Three-electrode potentiostat consisting of four operational amplifiers; 3. NB-IoT & GNSS module; 4. Power management module; 5. SD Card module; 6. Multiplexer and electrode interface; 7. Human-computer interaction module, including keyboard and screen. (**b**) Schematic diagram of circuit structure of the PMCED constant potential instrument. (**c**) Picture of PMCED.

The PMCED is designed based on four aspects: low cost, small size, easy operation, and data sharing. It consists of a three-electrode potentiostat, a multi-channel selector, a wireless communication module and positioning module, and a human-computer interaction unit on a compact 95 mm × 70 mm size printed circuit board (Figure 1a). The hardware cost of the PMCED is very low—only $30, as shown in Table 1.

### 2.1. Potentiostat

The potentiostat is used to control the potential between the working electrode (WE) and the reference electrode (RE) and measure the current formed by the redox reaction at the working electrode (Figure 1b). It is composed of four operational amplifiers (AD8608) and operates under the control of an analog-to-digital converter (ADC) and digital-to-analog converter (DAC) inside the microcontroller (MCU, STM32F103). The DAC provides the potential control signal, and the signal noise is attenuated by a filter composed of two operational amplifiers. The potential between the RE and WE is modulated under the control signal. The DAC provides a voltage scanning range of ±1.5 V. At 3.3 V reference voltage, the maximum quantization error of a 12-bit DAC is only 0.4 mV, which satisfies the generation of most small-waveform signals. In addition, the device emphasizes the accurate measurement of small peaks with a high background current. The transimpedance amplifier (TIA) connected in series with the WE completes the task of converting a high-resistance electrochemical cell into a low-resistance voltage source. Due to the high input resistance of operational amplifiers, the measured current almost completely flows through the feedback resistor *R_f_* across the reverse input and output terminals and is converted into a voltage signal. It should be noted that in the electrochemical cell, the equivalent capacitance and *R_f_* will form unnecessary poles in the loop gain of the amplifier, resulting in the instability of the closed-loop circuit. Therefore, we are paralleling a small capacitor *C_f_* and *R_f_*, and adding zeros to the feedback factor to increase stability. The use of the TIA not only greatly reduces the introduction of noise into the measurement process but also allows the use of the ADC to obtain current signals. The jumper high-precision 10 kOhm resistor not only sets the appropriate current sensitivity but also brings accurate, current gain. In addition, the step size of the ADC becomes the main factor in limiting the resolution of low current. The 12-bit ADC used by the PMCED can distinguish the current at a nA level under 10^4^ gain, as shown in Equation (1). The performance and volume are balanced by using an MCU on-chip ADC and DAC to drive the constant potential meter.

(1) $$\frac{V_{ref}}{2^{12}}\times \frac{1}{R_{G}}=I_{\min}$$

wherein, *V_ref_* is the reference voltage of ADC, and *R_G_* is the absolute value of current voltage conversion gain.

### 2.2. Multiplexer

The multiplexer (MUX, ADG708BRUZ) allows 4 electrodes to multiplex the potentiostat. The low-cost commercial screen-printed carbon electrode (SPCE, Ningbo, China, Xenletg studio) can be directly inserted into the electrode slot for electrochemical measurement. The reference electrode of SPCE is Ag/AgCl, and its counter electrode and working electrode are carbon electrodes. The design of a multi-channel selector is helpful for rapid multi-sample measurement in the field and can also provide a platform for the use of sensor arrays.

### 2.3. User Interface

The main elements of the user interface, keyboard, and screen also appear in the PMCED. We use a low-cost combination of monochrome OLED panels and buttons. At the same time, a complete set of interactive graphical interfaces is designed for the PMCED, which is convenient for users to skillfully use the device with minimum learning. The interface includes an electrode channel selection, electrochemical method selection, parameter setting, and inspection progress information viewing. The data obtained on-site can be shared with external devices through wireless interfaces. The PMCED combines low-power Bluetooth (CH9140) and NB-IoT modules (Quectel BC20).

In addition, as a portable device, the functional duration and weight of the PMCED are also considered. The overall power consumption of the electrochemical analysis is about 165 mW. When the NB-IoT module is in active mode, the power consumption of the module is about 390 mW, and the overall working power is 560 mW. In standby mode, the power consumption of the PMCED can be as low as 60 mW. The assembled high-polymer lithium battery gives consideration to both weight and battery capacity. The energy storage capacity of 3000 mAh brings about 90 h of continuous operation. At the same time, the weight of this battery is controlled within 100 g.

## 3. Wireless Communication System

Limited by the storage and computing power of portable devices, on-site detection data needs to be shared with peripheral or remote databases for the convenience of researchers. Therefore, the PMCED combines Bluetooth and NB-IoT modules to build a wireless communication system. As a low-cost, small-sized air interface, Bluetooth can directly establish a link within a short distance to complete data interaction. As a complement, NB-IoT plays a role in wide-area communication, which relies on cellular networks to establish connections in a wider coverage area. It is worth noting that Quectel BC20 is a multi-purpose module; in addition to NB-IoT services, it can also provide geographic location information through the Global Positioning System (GPS) and Beidou Satellite Navigation System (BDS). From this, the PMCED is able to acquire sampling locations for measurement samples.

**Figure 2:**
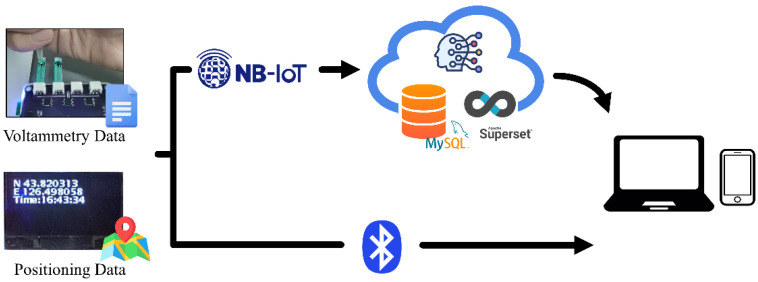
Wireless communication system built around the PMCED. The PMCED can not only exchange data over a short distance with smartphones/PCs through Bluetooth, but also upload data to the cloud through the NB-IoT network to achieve data transmission over a long distance.

Figure 2 shows the overall structure of two wireless interaction modes built around the PMCED. After the PMCED completes the electrochemical detection of the analyte, the voltammetric data, longitude, latitude information, and time information are stored in the SD card. These data can be shared with nearby smartphones and personal computers through Bluetooth, and the measurement results obtained in remote areas can also be uploaded to the cloud server for processing through the NB-IoT network. The cloud server has sufficient storage space and computing power to complete data management and calculation of stoichiometric models. MySQL, an open-source database, provides reliable and secure data management services. Apache Superset provides Web services as a visualization platform. It connects to the database and displays the analysis results with rich visual effects. Users can remotely access the data using the network. The researchers, with permission, can obtain the original data of on-site sampling from the database for more purposes.

## 4. Data Analysis and Processing

**Figure 3:**
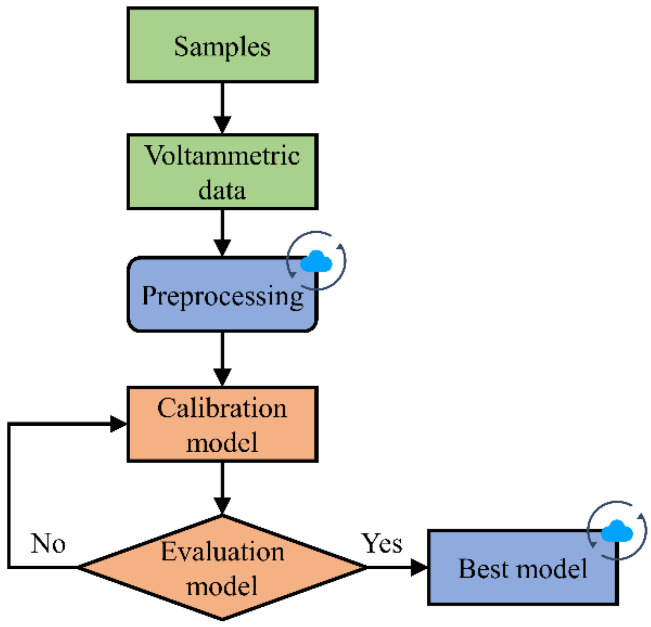
The processing flow of voltammetric data obtained by the PMCED. The cloud tag indicates a function carried out on the cloud server.

The voltammetric data obtained by the PMCED need to be converted into the indicator of the analyte with the help of mathematical methods. Therefore, a data analysis process, including preprocessing and a chemometric model, is constructed. The main purpose of preprocessing is to smooth the original voltammetric data, using a Butterworth low-pass filter to reduce the high-frequency line noise contained in the voltammetric data and improve the signal-to-noise ratio of the data. Data preprocessing is a flexible and optional process, and its usage depends on the degree of help to model analysis. The next step after data preprocessing is the use of calibration models. Chemometrics tools that can perform multivariate calibration are used to process voltammetric data and quantify analyte indicators. Partial least squares regression (PLSR), a classical statistical regression model, provides an effective way for quantitative analysis. It identifies potential variables that can represent the relationship between variables and response variables by establishing the correlation between them and then completes regression in the dimensional space of potential variables. Finally, the evaluated chemometrics model is deployed in the cloud to calculate and transform the analysis results. Figure 3 shows the process of data processing and the model used after the PMCED obtains the data.

Butterworth low-pass filter design and PLSR regression analysis in the processing flow were completed using the scipy and sklearn toolkits, respectively.

The evaluation indexes of model performance include determination coefficient (*R*^2^) and root mean square error (RMSE). *R*^2^ is defined by Equation (2).

(2) $$R^{2}=1-\frac{\sum_{i=1}^{n}{\left(y_{i}-{\hat{y}}_{i}\right)}^{2}}{\sum_{i=1}^{n}{\left(y_{i}-\bar{y}\right)}^{2}}$$

wherein $\bar{y}$ represents the average of the true value of y, for which $\hat{y}$ is the predicted value. When the prediction model does not make any mistakes, the maximum value of *R*^2^ is 1. When the model is equal to the benchmark model, the *R*^2^ value is 0.

Root mean square error (RMSE) is a typical indicator formula of a regression model, such as (3), which is used to indicate how much error the model will produce in prediction. For larger errors, the weight is higher.

(3) $$RMSE=\sqrt{\frac{1}{n}\sum_{i=1}^{n}{\left(y_{i}-{\hat{y}}_{i}\right)}^{2}}$$

wherein, y is the actual value, $\hat{y}$ is the predicted value, and *n* is the number of pieces of data used for calculation.

## 5. Results and Discussion

### 5.1. Electrochemical Performance of the PMCED

**Figure 4:**
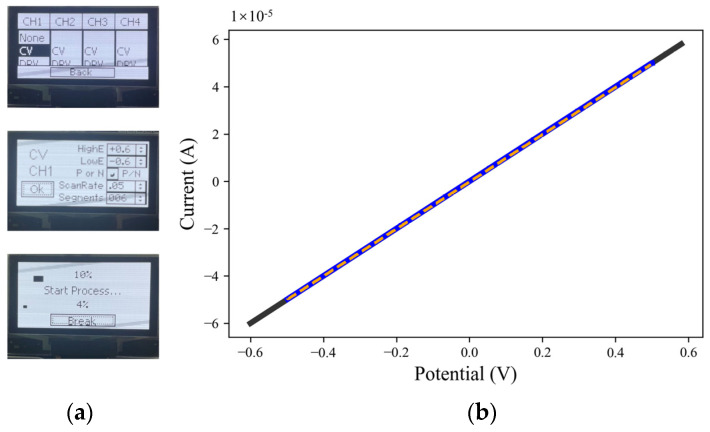
Operation of the PMCED. (**a**) The human–computer interface for selecting the method to be used and setting parameters, and viewing of the operation progress at the same time. (**b**) The volt-ampere curves of the PMCED and CHI760E at 10 kOhm resistors were compared. Black is the ideal 10 kOhm curve, blue is the curve obtained using the CHI760E test, and orange dotted line is the test curve obtained by PMCED.

The PMCED has an interactive graphical interface with a clear hierarchy. Users can quickly select the electrochemical analysis method and set parameters for each channel through key input and feedback from the interactive graphical interface without relying on any additional device [31,32]. Figure 4a shows the interactive interface for electrochemical settings. Users can select one of the two electrochemical methods (CV and DPV) for each channel and then set specific parameters, respectively. The completion of sample detection can also be obtained on the graphical interface. The progress bar at the top indicates the completion of detection for the current channel, and the progress bar at the bottom indicates the progress of the whole detection process. The PMCED provides a simple and clear human–computer interaction mode to help the electrochemical analysis device play a better role in on-site testing.

The potential holding and current measuring performances of the three-electrode potentiostat are shown with the measurement of resistors. The CE and RE are shorted, and then both ends of the 10 kOhm resistors are connected to the RE and WE, respectively, to scan in the potential range of −0.5 V to 0.5 V. Figure 4b shows that the volt-ampere characteristic curve obtained by the PMCED coincides with the ideal straight line height, and the slope of the curve is 1 × 10^−4^. The PMCED constant potential meter can output accurate potential and complete an accurate current measurement at the same time.

**Figure 5:**
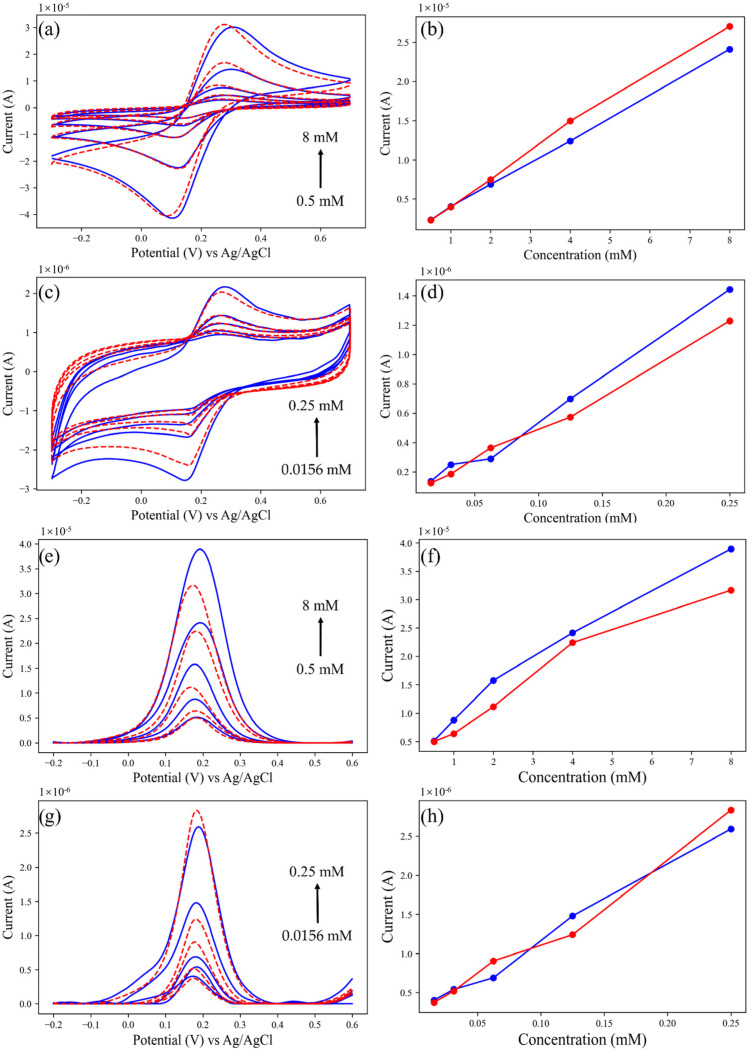
The PMCED and commercial potentiostat CHI760E measure potassium ferricyanide (0.0156–8 mM) at screen-printed electrode—results comparison. Red is the measurement result of the CHI760E, and blue is the measurement result of the PMCED. (**a**) Cyclic voltammogram of potassium ferricyanide at 0.0156–0.25 mM concentration. (**c**) Cyclic voltammogram of potassium ferricyanide at 0.5–8 mM concentration. (**e**) Differential pulse voltammetry of potassium ferricyanide at 0.0156–0.25 mM concentration. (**g**) Differential pulse voltammetry of potassium ferricyanide at 0.5–8 mM concentration. (**b**,**d**,**f**,**h**) are peak current comparison graphs, respectively.

Figure 5 shows the voltammogram of the PMCED and commercial electrochemical workstation (CHI760E, Shanghai CH instruments) using CV and DPV methods to detect ferricyanide in a concentration range of 0.0156 mM to 8 mM. Table 2 shows the values of the oxidation peak currents. In the CV comparison, the two devices obtained very similar cyclic voltammograms. The oxidation peak of the PMCED at 8 mM and 4 mM ferricyanide is lower than that of the CHI760E, which may be attributed to the attenuation of the filter when removing the noise in the oxidation process. In cyclic voltammetric assays for low concentrations of ferricyanide (0.0156–0.125 mM), the oxidation and reduction peak shapes measured by the two methods were consistent. It can be seen from the comparison of the oxidation peak that the PMCED has the same performance as the commercial electrochemical workstation in the detection of low current (the measuring current can reach the level of 100 nA). Figure 5e,g show the voltammogram obtained using the DPV method. The oxidation peaks measured by the PMCED are almost higher than the CHI760E, although the peak height has a good linear relationship with the concentration of ferricyanide. This difference mainly comes from the difference in current sampling time points between the PMCED and CHI760E. In the case of small-current measurement, the deviation caused by different sampling times during the attenuation of the non-faradaic current is significantly reduced. As can be seen from Figure 5h, the peak current of potassium ferricyanide at 0.0313 mM and 0.0156 mM concentration is almost the same.

### 5.2. Sample Processing and Chemometric Analysis

**Figure 6:**
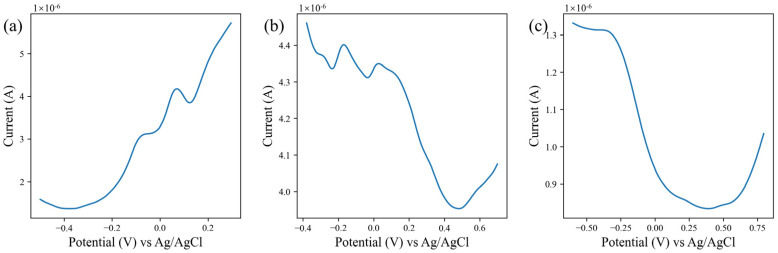
Differential pulse voltammetry of water sample. (**a**) Voltammetric diagram on AgNDs-SPE. (**b**) Voltammetric diagram on AuNPs-SPE. (**c**) Voltammetric diagram on rGO-SPE.

The PMCED was used to test water samples at different locations in Jilin, China, and test data from 18 water samples were obtained. The water sample was detected using the electrode array formed by the screen-printed electrode modified with nanomaterials [33]. This sensor array consists of gold nanoparticles (AuNPs), silver nanodendritic structures (AgNDs), and reduced graphene oxide (rGO)-modified screen-printed electrodes (SPEs). An amount of 100 μL of the water sample was dropped on the electrode area of each screen-printed electrode. With the help of a multi-channel selector, each electrode in the array worked under its own appropriate working parameters. The detailed parameters of DPV were: incremental potential 4 mV, pulse amplitude of 50 mV, and pulse width of 20 ms. The scanning range of AgNPs-SPE was −0.5 to 0.3 V; AuNPs-SPE was −0.38 to 0.7 V; rGO-SPE was −0.6 to 0.8 V. In order to improve the data quality of voltammogram, a Butterworth low-pass filter was used to reduce the noise in the original data. For AgNDs-SPE, AuNPs-SPE, and rGO-SPE, the cut-off frequencies of 0.2 rad/s, 0.14 rad/s, and 0.03 rad/s are set, respectively, for different levels of noise. The result after smoothing is shown in Figure 6.

After data preprocessing, the classical PLSR is used to establish the voltammogram and the regression model of COD_Mn_ in the samples. The results of COD_Mn_ analyzed by PMCED are compared with the results from a water quality test station using the traditional sodium oxalate titration method. Table 3 shows the performance of PLSR on the test set when the number of hidden variables is 4. Recovery (%) is defined by the equation [34] “Recovery (%) = A/B × 100%” (A is the concentration measured by our method. B is the concentration measured by the water quality test station). The PMCED showed good analysis results.

### 5.3. Cloud Computing and Visualization

**Figure 7:**
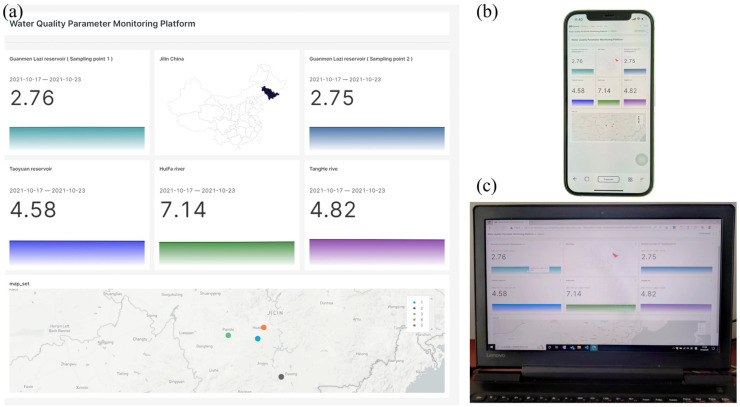
Visualization platform deployed on the cloud server. (**a**) Dashboard of the visualization platform. (**b**) Access the visualization platform through smartphones. (**c**) Access the visualization platform through a personal computer.

The PMCED interacts with the cloud through the NB-IoT network. The widely covered NB-IoT network can establish a reliable data transmission path in a wider range to ensure high-quality data transmission after on-site detection in a remote area. The original voltammetric data, geographical location, and time information are transmitted to the Baidu Cloud Server through the NB-IoT network. The cloud server runs a custom python script and continuously monitors the data-receiving port. At the same time, it preprocesses the original data and undertakes the calculation of the PLSR model. Finally, the geographic coordinates, time information, and COD_Mn_ quantification values of the detected water samples are imported into the MySQL database of the server for centralized management and sharing. The cloud server also deploys a SuperSet data visualization platform, which periodically obtains data from the database and displays it. Figure 7a shows the layout of the cloud visualization platform. Each sub-column is the COD_Mn_ value of water samples at each testing point and also contains time information and historical testing curves. The geographic coordinate information is accurately projected onto the map interface, which can clearly show the location of each sampling point in the geographic space. Users can access the cloud data visualization platform (Figure 7b,c) via smartphones or computers at any time and anywhere under the coverage of the network to view the COD_Mn_ value of natural water from the sampling points. Cloud computing and cloud visualization provide flexible and convenient access while reducing the pressure on the PMCED for local computing.

## 6. Conclusions

This work establishes a portable multi-channel electrochemical device with the capability for low current detection, which satisfies the precise measurement of tiny current peaks on the high background current of water samples in the field. The PMCED provides an easy-to-operate graphical interactive interface, which can independently and conveniently select electrochemical methods and set parameters. In addition, the combination of Bluetooth and NB-IoT wireless connection modes brings data transmission means of short-distance data interaction and remote access to a cloud server. The system has built an electrochemical data storage and analysis process in the cloud, and the visualization platform can show the analysis results to visitors. In the application of detecting COD_Mn_ in natural water, the portable device has tested water samples on-site. Finally, the geographic location of each sampling point and the COD_Mn_ in the water sample are displayed on the web page. The above results show that the portable electrochemical analysis system is an ideal platform for on-site measurement and analysis.

## Figures and Tables

**Table 1:** Cost of materials for the PMCED.

Name	Description	Quantity	Unit Price ($)	Total ($)
STM32F103RC	MCU	1	2.8	2.8
AD8608	Quad Op-amp	1	3.0	3.0
REF196	3.3 V Reference	1	1.4	1.4
AMS1117	3.3 V Regulator	1	0.14	0.14
CH9140	BLE Module	1	1.4	1.4
BC20	NB-IoT & GNSS Module	1	4.7	4.7
TXS0108EPWR	Voltage translator	1	0.72	0.72
TP5400	Li-ion battery management	1	0.07	0.07
ADG708BRUZ	8-channel multiplexers	3	2.15	6.45
CH340N	USB to TTL	1	0.28	0.28
Passive elements	Resistors and capacitors	36	0.02	0.72
USB connector	USB type-C connector	1	0.14	0.14
Keys	Button	8	0.0175	0.14
OLED	Monochrome display panel	1	2	2
Lithium battery	3000 mAh battery	1	4.3	4.3
Total Cost:				28.26

**Table 2:** The oxidation signal of various concentrations of the [Fe(CN)_6_]^3−^ solution measured with CHI760E or PMCED via CV and DPV.

Concentration (mM)	Peak Current (μA)			
	CV		DPV	
	PMCED	CHI760E	PMCED	CHI760E
0.0156	0.137	0.125	0.403	0.373
0.0313	0.251	0.185	0.541	0.519
0.0625	0.290	0.365	0.689	0.904
0.125	0.698	0.573	1.480	1.240
0.250	1.443	1.230	2.591	2.834
0.500	2.324	2.306	5.134	5.021
1.00	4.040	3.973	8.782	6.411
2.00	6.853	7.464	15.762	11.138
4.00	12.392	14.952	24.137	22.405
8.00	24.120	27.013	38.935	31.649

**Table 3:** COD_Mn_ Results of 6 water samples detected by the PMCED and water quality test station.

	PMCED Detected (mg/L)	Water Quality Test Station Detected (mg/L)	Recovery (%)
Sample 1	4.71	4.89	96.3
Sample 2	2.98	2.75	108.4
Sample 3	3.49	2.75	126.9
Sample 4	4.30	4.62	93.1
Sample 5	6.51	6.99	93.1
Sample 6	4.99	4.89	102
RMSE (mg/L): 0.44		*R*^2^: 0.91	

## Data Availability

Not applicable.
